# American Society for Enhanced Recovery (ASER) and Perioperative Quality Initiative  (POQI) joint consensus statement on perioperative fluid management within an enhanced recovery pathway for colorectal surgery

**DOI:** 10.1186/s13741-016-0049-9

**Published:** 2016-09-17

**Authors:** Robert H. Thiele, Karthik Raghunathan, C. S. Brudney, Dileep N. Lobo, Daniel Martin, Anthony Senagore, Maxime Cannesson, Tong Joo Gan, Michael Monty G. Mythen, Andrew D. Shaw, Timothy E. Miller, For the Perioperative Quality Initiative (POQI) I Workgroup

**Affiliations:** 1 Departments of Anesthesiology and Biomedical Engineering, Divisions of Cardiac, Thoracic, and Critical Care Anesthesiology, UVA Enhanced Recovery after Surgery (ERAS) Program, University of Virginia School of Medicine, Charlottesville, VA USA; 2 Department of Anesthesiology, Duke University Medical Center, Durham, NC 27710 USA; 3 Duke University and Durham VA Medical Center, Durham, NC USA; 4 Gastrointestinal Surgery, National Institute for Health Research Nottingham Digestive Diseases Biomedical Research Unit, Nottingham University Hospitals and University of Nottingham, Queen’s Medical Centre, Nottingham, NG7 2UH UK; 5 Division of Surgery and Interventional Science, University College London, Royal Free Hospital, London, NW3 2QG UK; 6 Anaesthetic Department, Royal Free Perioperative Research Group, Royal Free Hospital, London, NW3 2QG UK; 7 Department of Surgery, University of Texas-Medical Branch at Galveston, Galveston, TX 77555 USA; 8 Department of Anesthesiology and Perioperative Medicine, University of California Los Angeles, Los Angeles, CA USA; 9 Department of Anesthesiology, Stony Brook University School of Medicine, Stony Brook, NY USA; 10 University College London Hospitals, National Institute of Health Research Biomedical Research Centre, London, UK; 11 Department of Anesthesiology, Vanderbilt University School of Medicine, Nashville, TN USA; 12 Division of General, Vascular and Transplant Anesthesia, American Society for Enhanced Recovery, Duke University Medical Center, Durham, NC 27710 USA

**Keywords:** Enhanced recovery pathway, ﻿Enhanced recovery, Fluids, Colorectal surgery, Crystalloids, Colloids, Goal-directed fluid therapy, Carbohydrate drink, Hemodynamics

## Abstract

**Background:**

Enhanced recovery may be viewed as a comprehensive approach to improving meaningful outcomes in patients undergoing major surgery. Evidence to support enhanced recovery pathways (ERPs) is strong in patients undergoing colorectal surgery. There is some controversy about the adoption of specific elements in enhanced recovery “bundles” because the relative importance of different components of ERPs is hard to discern (a consequence of multiple simultaneous changes in clinical practice when ERPs are initiated). There is evidence that specific approaches to fluid management are better than alternatives in patients undergoing colorectal surgery; however, several specific questions remain.

**Methods:**

In the “Perioperative Quality Initiative (POQI) Fluids” workgroup, we developed a framework broadly applicable to the perioperative management of intravenous fluid therapy in patients undergoing elective colorectal surgery within an ERP.

**Discussion:**

We discussed aspects of ERPs that impact fluid management and made recommendations or suggestions on topics such as bowel preparation; preoperative oral hydration; intraoperative fluid therapy with and without devices for goal-directed fluid therapy; and type of fluid.

## Consensus statements

### Prior to surgery

1. We recommend unrestricted access to clear fluids for oral intake up to 2 h before the induction of anesthesia to maintain hydration while minimizing the risk of aspiration.
2. We recommend that the clear fluid used to maintain oral hydration contain at least 45 g of carbohydrate to improve insulin sensitivity (except in type I diabetics due to their insulin deficiency state). We suggest that complex carbohydrate (e.g., maltodextrin) be used when available.
3. We recommend that clinicians avoid administration of intravenous fluids to replace preoperative “fluid losses” in patients who received iso-osmotic bowel preparation provided there was unrestricted intake of clear fluids for up to 2 h before the induction of anesthesia. There is no evidence that iso-osmotic mechanical bowel preparation leads to adverse effects on preoperative volume status.
4. We recommend against the use of hyper-osmotic or hypo-osmotic bowel preparations prior to surgery since there is no benefit relative to iso-osmotic bowel preparation and there may be adverse effects on preoperative volume status.

### During and after surgery

5. We recommend the application of a hemodynamic framework to guide clinical decision-making during surgery. We have developed such a framework and suggest that the use of intraoperative goal-directed fluid therapy (GDFT) is likely to be safe in the majority of patients undergoing major colorectal surgery. GDFT has little risk, and the use of advanced hemodynamic monitoring equipment may enhance clinical decision-making when compared with the use of conventional monitors.
6. We suggest that the advanced hemodynamic monitoring equipment used to guide clinical decision-making intraoperatively be selected based on a combination of surgical patient and institutional factors since such monitoring can minimize both hypovolemia (by promoting therapy in volume responders) and hypervolemia (by restricting therapy in non-responders).
7. We recommend that in isolation, intraoperative oliguria should not trigger fluid therapy, as low urine output is a normal physiologic response during surgery and anesthesia. We also recommend that intraoperative oliguria be investigated and that absolute (as opposed to relative) hypovolemia be ruled out.
8. We recommend that intraoperative and postoperative anuria warrant immediate attention since anuria is pathological.
9. We recommend that fluid management strategies focus on the following: first, identifying if there is a clinical problem that can be solved by fluid therapy and then identifying what fluid and how much is appropriate. Rather than treating every instance of abnormal hemodynamic values (displayed by conventional or advanced monitors), clinicians must establish causation based on available information about the patient and clinical context.
10. We recommend that therapy attempt to reverse the most likely cause of a hemodynamic derangement. Absolute hypovolemia may or may not be responsible for observed hemodynamic abnormalities. For instance, stroke volume variation above 13 % soon after the induction of anesthesia and with the institution of mechanical ventilation should prompt consideration of vasodilation (relative hypovolemia) rather than as the cause of fluid responsiveness. The patient may hence require vasoconstrictors rather than bolus fluid therapy provided clear fluids have been consumed preoperatively and iso-osmotic bowel preparation has been used.
11. We recommend the use of buffered isotonic crystalloids for the treatment of hypovolemia in patients undergoing colorectal surgical procedures. We acknowledge that the restrictions on the use of starch solutions are based on extrapolations from the critical care literature.
12. We suggest that patients tolerating fluids orally after surgery be given unrestricted access to such fluids as this increases patient satisfaction and as it is likely that intravenous fluid administration offer no added benefit.
13. We suggest that the hemodynamic framework utilized intraoperatively be extended into the postoperative period to the extent possible, in situations where patients might benefit from such postoperative monitoring (high-risk patients or those with significant blood loss or complications during surgery).

## Background

Outcomes such as complication rates, readmissions, and length of stay may be highly variable across different centers conducting colorectal surgery (Cohen et al. 2009). Enhanced recovery pathways (ERPs), initially led in Europe (by the surgeon Henrik Kehlet), were developed in the 1990s in an effort to reduce such variability (Kehlet 1997). ERPs generally share certain core features but also have subtle differences across and even within sites (reflecting unique institutional needs, capabilities, and resource availability). Most ERPs focus on setting patient expectations and involving the patient in their own care pathway for fast recovery, avoiding prolonged preoperative restriction of fluid intake, avoidance of empirical intravenous fluid loading, minimization of systemic opioid use, and early postoperative ambulation. In several studies examining effects of ERP implementation (versus data prior to implementation), an average reduction in length of stay of 3 days appeared to result in over 3000 subjects across several institutions and in a variety of surgical procedures (Thiele et al. 2015a). Meta-analyses on ERPs in colorectal surgical procedures found similar reductions in length of stay (~2.5 days) without an increase in readmission rates (Zhuang et al. 2013; Varadhan et al. 2010).

Retrospective and prospective studies of ERPs in colorectal surgery have typically examined “bundled” interventions making it difficult to estimate the relative value of specific elements related to perioperative fluid management. Outside the context of an ERP, investigators have defined outcomes following “liberal” and “restrictive” fluid strategies during colorectal surgery. However, there is no shared definition of what amount constitutes either (Chappell et al. 2008). Calculations of intraoperative fluid deficits during colorectal surgery have, prior to ERP, included so-called “third space” losses and perioperative fluid therapy was guided by static indicators of volume status. A recent comprehensive review summarized this as follows: “Research suffers from a lack of standardization…Investigators have normally named their traditional regimen the standard group and compared it with their own restrictive ideas… A restrictive regimen in one study is often designated as liberal in another setup…This shortcoming prevents even promising results from impacting daily clinical routine and makes any pooling of the data impossible*.*” (Chappell et al. 2008) Thus, several specific questions, related to fluid therapy, remain. The “fluids” subgroup within the first Perioperative Quality Initiative (POQI) sought to define and answer important questions related to perioperative fluid management in patients undergoing colorectal surgery within the context of an ERP.

## Methods/design

Applying a modified Delphi method, designed to use the collective expertise of a diverse group of experts to answer clinical questions, we achieved consensus on several topics related to perioperative fluid management in patients undergoing colorectal surgery within the context of an ERP.

### Expert group

An international group of authorities, with specific content area expertise (based on the conduct of research and education in this area), was invited to participate. In total, 32 experts from around North America and Europe met in Durham, NC, on March 4–5, 2016, to iteratively discuss the evidence supporting enhanced recovery paradigms and develop consensus statements with practical recommendations for clinicians.

### Process

A list of relevant questions was collectively developed and circulated electronically prior to the meeting. Based on literature searches performed by members, questions were formulated. In the first plenary session, the POQI perioperative fluid management subgroup presented these questions to the entire POQI workgroup, to receive feedback and assistance in refining the questions. The subgroup then worked together to formulate answers to these questions, supported by evidence when available and by expert opinion when no clear evidence was available. These were presented in the second plenary session. After receiving feedback, the subgroup refined a series of consensus statements, which was then reviewed with and modified by the entire POQI group in the final plenary session. This manuscript is based on these multiple rounds of feedback from *all* the experts present at the first POQI meeting.

## Results

Based on both discussions (held prior to the conference) and the literature (identified by the participants), the following questions were considered most relevant to perioperative fluid management before, during, and after colorectal surgery within an ERP:

### Prior to surgery

(i) What are the effects of preoperative oral intake of clear solutions (containing complex versus simple carbohydrates) up to 2 h prior to the induction of anesthesia?
(ii) Does mechanical bowel preparation contribute to preoperative hypovolemia?

### During and after surgery

(iii) Is urine output a valid indicator of perioperative fluid needs?
(iv) Is there a rational approach to intraoperative fluid management based on the current evidence?
(v) Which types of fluids should be used intraoperatively?
(vi) How do variations in surgical and anesthesia technique affect intraoperative fluid management?
(vii) How should fluid therapy be managed postoperatively?

### (i) What are the effects of preoperative oral intake of clear solutions (containing complex versus simple carbohydrates) up to 2 h prior to the induction of anesthesia?

It has known that both simple (e.g., glucose) and complex (e.g., maltodextrin) carbohydrate-containing solutions prevent protein catabolism following exercise (Borsheim et al. 2004; Roy et al. 1997). Whether this is true in the perioperative period has not, until recently, been known. In animals, oral maltodextrin solution prior to sham surgery reduces protein catabolism versus fasting (with ad libitum water) (Luttikhold et al. 2013). A trial comparing a high to low maltodextrin beverages before surgery found stable post-surgical protein balance in the high but negative whole-body protein balance in the low group (Svanfeldt et al. 2007). Such data suggest that preoperative oral intake of clear solutions containing certain carbohydrates may prevent perioperative protein catabolism. Larger studies are needed to better examine impact on meaningful clinical outcomes such as length of stay or surgical complications. In a recent Cochrane Review including 1976 participants in 27 trials comparing preoperative carbohydrate loading with placebo, where preoperative carbohydrate loading was defined as the intake of at least 45 g of carbohydrates within 4 h prior to surgery, a trend towards improved postoperative insulin resistance was demonstrated (as measured by the Homeostatic Model Assessment of Insulin Resistance (HOMA-IR)) (Smith et al. 2014). On the other hand, a different meta-analysis showed that although there was a tendency toward reduction of postoperative insulin resistance, preoperative carbohydrate loading made no difference to the rates of postoperative complications (Awad et al. 2013).

In non-diabetic colectomy patients, it appears that upwards of 25 % are at risk for postoperative hyperglycemia with associated risks of SSI and mortality, presumably from acute insulin resistance (Kwon et al. 2013). Measuring insulin sensitivity with the hyperinsulinemic euglycemic clamp method, carbohydrate loading (as compared with placebo or fasting) demonstrated a trend towards increased postoperative insulin sensitivity (sensitivity difference 0.24 to 1.29, *p* = 0.0046) (Smith et al. 2014). Additionally, Cochrane analysis identified a reduction in length of stay of 0.30 days with carbohydrate loading versus fasting but not versus placebo (−0.12 days, 95 % confidence interval −0.38 to 0.12 days) (Smith et al. 2014). Much (but not all) of the data on preoperative carbohydrate loading was based on the use of maltodextrin-containing solutions. Direct comparisons with more readily available simple sugar containing solutions (e.g., glucose) have not been made. However, there are significant data suggesting the negative impact of a high versus low glycemic index meal on the response of glucose, insulin, and glucagon (Harbis et al. 2004).

Overall, based on the low risk of harm, potentially improved nitrogen balance, and better insulin sensitivity following colorectal surgery, we recommend the oral intake of carbohydrate-containing solutions prior to surgery and suggest that solutions containing complex carbohydrates be used when available. We acknowledge that cost and convenience may be barriers to the use of such solutions. It is worth noting that in patients with type I diabetes, provision of such solutions may offer no benefit over electrolyte-containing water with the possible exception of improved nitrogen balance. Lastly, in order to minimize potential risks of aspiration, as detailed in the guidelines issued by the American Society of Anesthesiologists (ASA), the oral intake of clear liquids should occur more than 2 h prior to the induction of anesthesia. ASA guidelines recommend modification of preoperative fasting on an individual basis in the presence of “gastroesophageal reflux disease, dysphagia symptoms, or other gastrointestinal motility disorders.” (American Society of Anesthesiologists C 2011)

### (ii) Does mechanical bowel preparation contribute to preoperative hypovolemia?

The traditional view, that mechanical bowel preparation (MBP) may lead to hypovolemia due to gastrointestinal losses prior to colorectal surgery, was supported by a study comparing ten subjects randomized to Picolax (magnesium citrate (a hyper-osmotic laxative) and sodium picosulfate (a stimulant)) versus not. There was significantly more orthostasis and tachycardia in the group that received the hyper-osmotic bowel preparation (Barker et al. 1992). In a subsequent trial comparing 41 patients receiving Picolax randomized to oral intake and protocoled intravenous fluid administration versus oral intake alone, there was more weight loss, hemoconcentration, and orthostasis (a highly specific, but relatively insensitive marker for hypovolemia and fluid responsiveness) in the group receiving no intravenous fluid (Sanders et al. 2001). Similar investigations on hyper-osmotic MBP with bisacodyl, sodium phosphate, and metoclopramide, followed by prespecified fluid intake over 3 days (in 12 healthy volunteers) revealed a median weight loss of 1.2 kg, decrease in exercise tolerance (median 9 % reduction in watts), but no changes in orthostasis (Holte et al. 2004). The small sizes of these studies, relative health of the volunteers, and the lack of surgery make it challenging to interpret these data, although weight loss (presumably due to fluid losses from the gastrointestinal tract) is incontrovertible.

In a clinical study of 19 patients undergoing laparoscopic colonic surgery after MBP (bisacodyl, polyethylene glycol 24 h preoperatively), the mean cardiac index was 2.66 L/m^2^ (normal range 3.5–5) based on transpulmonary thermodilution, after induction of anesthesia. The authors concluded that hypovolemia was likely to be present (Junghans et al. 2006). Unfortunately, the lack of control comparators makes attribution of changes in cardiac index to MBP difficult. The effects of induction of general anesthesia cannot be controlled for. In a meta-analysis of prospective trials studying MBP for colorectal surgery examining the risk of “cardiac events,” MBP was associated with an increased incidence of such events (2.9 versus 4.6 % among 2472 patients included in the meta-analysis) (Gravante et al. 2008).

Modern MBP techniques typically utilize iso-osmotic agents, which in theory do not produce dehydration (no osmotic shift in fluids toward the bowel lumen). When combined with the emphasis on intake of clear fluids up to 2 h before surgery (see question i) in compliance with the ASA Fasting Guidelines, concerns related to the impact of MBP on volume status are minimal. There is no need for fluid therapy to treat presumed fluid losses from iso-osmotic MBP and starvation. The shift in practice away from empiric administration of fluid therapy toward therapy based on the detection of “fluid responsiveness” (defined as a specified increase in cardiac output following fluid administration, typically at least by 10 %) has further diminished arbitrary preoperative intravenous hydration. If clinicians are able to rapidly identify hypovolemia intraoperatively, excessive preoperative fluid losses can be detected and managed objectively. Since it has not been established that iso-osmotic MBP predisposes patients to hypovolemia, and since clinicians can identify patients in whom MBP might have produced excessive fluid losses promptly (utilizing methods such as respiratory variation in the plethysmography preoperatively (Tsuchiya et al. 2010), we recommend against empiric pre-emptive intravenous fluid therapy to correct MBP-induced hypovolemia. In summary, while some data have previously suggested that hyper-osmotic MBP leads to dehydration before surgery (with increased risk of perioperative adverse cardiac events), there is no evidence that iso-osmotic MBP leads to any hemodynamic perturbations or increased cardiac risk among patients allowed unrestricted access to clear fluids for oral intake prior to surgery.

### (iii) Is urine output a valid indicator of perioperative fluid needs?

Traditionally, urine output has been viewed as an indicator of the adequacy of kidney perfusion. Anuria is abnormal and should always be a cause for concern warranting prompt investigation. However, oliguria, defined as urine output less than 0.5 mL/kg/h by the Kidney Disease: Improving Global Outcomes (KDIGO) group, is more challenging to interpret as abnormal, especially when other indicators of overall tissue perfusion are normal (Section 2: AKI Definition. Kidney Int Suppl 2012).

For instance, it is now understood that the release of vasopressin (antidiuretic hormone) is a natural response to anesthesia and surgery. The resorptive actions of vasopressin on the collecting duct in nephrons lead to the retention of water with accompanying oliguria—this may not indicate organ dysfunction (Cochrane et al. 1981). In 1984, a study from the Cleveland Clinic cast doubt on the utility of perioperative oliguria as an indicator of tissue hypoperfusion requiring fluid therapy. Among 137 patients undergoing aortic reconstruction surgery, mean intraoperative urine output or lowest intraoperative urine output had no relationship to changes in postoperative BUN or creatinine levels (Alpert et al. 1984). More recently, in a meta-analysis (of 1594 patients across 15 studies) examining whether intraoperative fluid restriction leads to perioperative acute kidney injury (AKI), there was a trend towards oliguria (OR 2.07, 95 % CI 0.97 to 4.44) but there was no difference in the incidence of AKI (OR 1.07, 95 % CI 0.60 to 1.92) (Egal et al. 2016a). Further support for the insensitivity of oliguria is provided by analysis of 1444 cases where the administration of less than or equal to 3 mL/kg/h of crystalloid during surgery was not associated with the development of AKI (Ahn et al. 2016). This study also found no difference in rates of oliguria and intraoperative urine output among patients that developed AKI versus those that did not (Ahn et al. 2016).

In contrast, there are data suggesting that high volumes of postoperative urine may indicate recovery and predict early readiness for discharge among patients undergoing colorectal surgery (Johnson et al. 2015). In order to study the effect of forced diuresis, Egal et al. analyzed the impact of oliguria reversal (i.e., targeting a specific urine output) on outcomes in a heterogenous population of 4825 patients in 28 studies undergoing GDFT. They found that while GDFT algorithms with a specific urine target did not reduce AKI compared to conventional fluid management (CFM) but that GDFT algorithms that ignored urine output did (Egal et al. 2016b). Additionally, there is some evidence in cardiac surgical patients that the administration of furosemide (presumably to reverse oliguria) leads to increased serum creatinine (Lassnigg et al. 2000). Such data suggest that while anuria is abnormal, oliguria is a normal “stress” response to surgery even conferring some clinical benefit. There are also data suggesting that the reversal of oliguria either attenuates the potential benefits of protocolized hemodynamic management strategies or even causes harm (Egal et al. 2016b). While much of this data—on urine output and renal function—comes from patients undergoing non-colorectal procedures (and in some cases, non-surgical patients), there are no prospective, randomized controlled trials comparing oliguria reversal (forced diuresis) to more conventional management in patients undergoing colorectal surgery. There is no evidence that urine output is a valid indicator of a need for fluid therapy in patients undergoing colorectal surgery. We recommend that low urine output, as an isolated abnormality, should not trigger fluid therapy and should trigger diagnostic efforts.

### (iv) Is there a rational approach to intraoperative fluid management based on the current evidence?

Minimally invasive devices, that reflect cardiac output in real time without pulmonary artery catheterization, have allowed the perioperative measurement of global blood flow. Fluid therapy guided by such devices may thus be based on “fluid responsiveness” where repeated administration of fluid boluses occurs when patients “respond” to fluids (by objectively increasing global blood flow). Minimally invasive cardiac output monitoring-guided fluid management has been studied in multiple randomized controlled trials in patients undergoing diverse procedures (Sinclair et al. 1997; Gan et al. 2002; Venn et al. 2002; Wakeling et al. 2005; Noblett et al. 2006; Chytra et al. 2007; Pillai et al. 2011; Jones et al. 2013; Ni et al. 2013), including abdominal surgery. The sum of these studies suggest that application of minimally invasive cardiac output monitoring can reduce the length of stay (Sinclair et al. 1997; Gan et al. 2002; Venn et al. 2002; Wakeling et al. 2005; Noblett et al. 2006; Chytra et al. 2007; Pillai et al. 2011; Jones et al. 2013; Ni et al. 2013).

Brandstrup et al. offered an alternative to perioperative minimally invasive cardiac output monitoring, by demonstrating that a “zero-fluid balance” approach was as effective in terms of length of stay as well as incident complications (Brandstrup et al. 2003). How could it be that the initial GDFT studies, as conducted by Gan et al., suggested significant benefit with minimally invasive cardiac output monitoring (reduced length of stay), but that more recent studies show that similar benefits can be accrued by paying meticulous attention to avoiding fluid overload and maintaining “zero balance” (without the use of additional devices) (Gan et al. 2002; Brandstrup et al. 2003)? These apparently “conflicting” results may be reconciled by understanding that clinical trials examine outcomes relative to a comparator group. Depending on the fluid management strategies in this control group, achieving near-maximal stroke volume with a GDFT approach either offers an advantage (when the alternative is empirically “liberal” therapy) or is equivalent (when the alternative is “restrictive”) (Chappell et al. 2008). Thus, a rational interpretation of evidence is that there is potential benefit from GDFT using minimally invasive cardiac output monitoring devices by both avoiding unnecessary fluid therapy in volume non-responders and avoiding inadequate fluid therapy and hypoperfusion in volume responders (Bellamy’s conceptual model for keeping patients “optimized”) (Bellamy 2006).

**Fig. 1:**
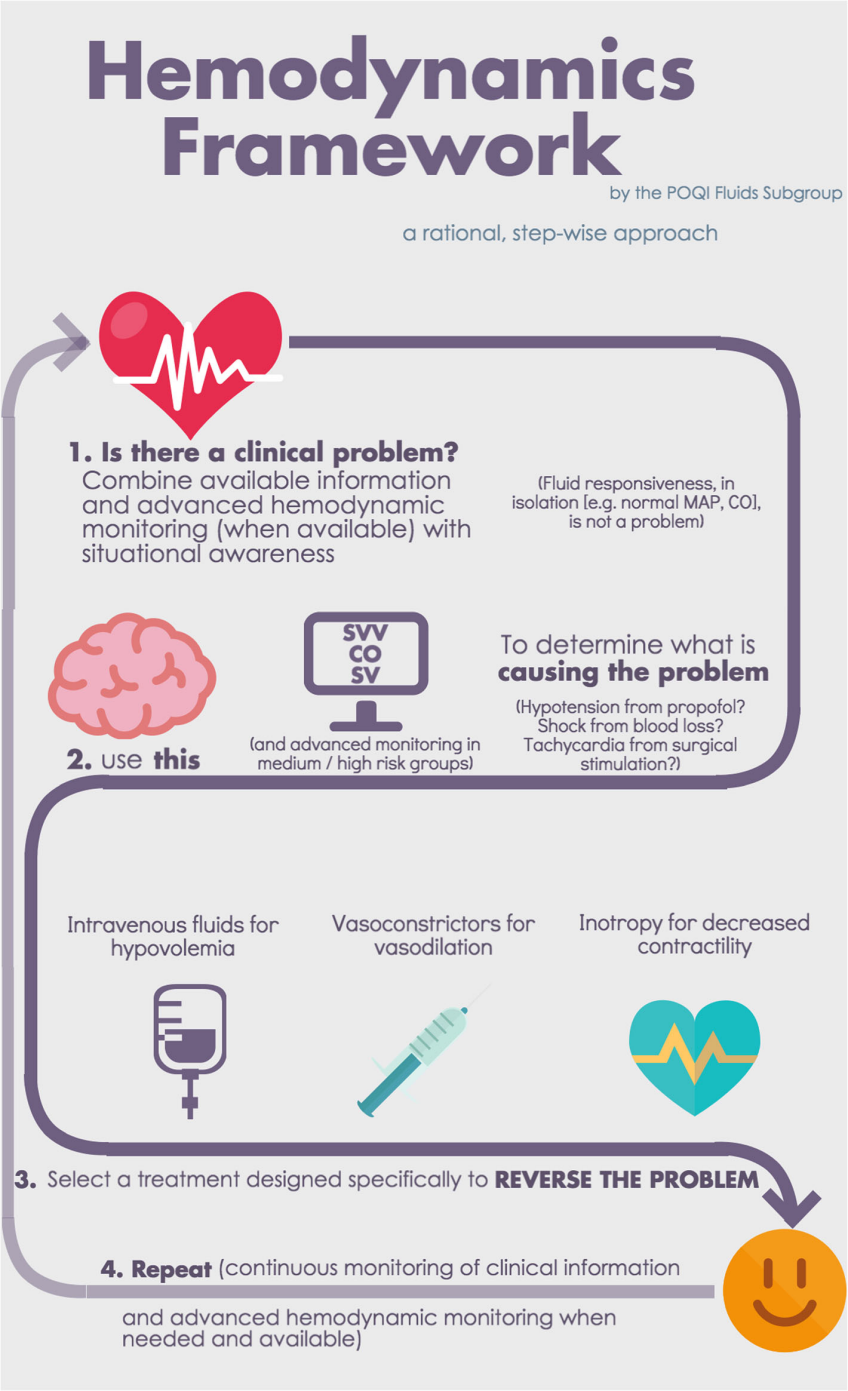
Suggested clinical framework for managing perioperative hemodynamics in patients undergoing colorectal surgical procedures

**Fig. 2:**
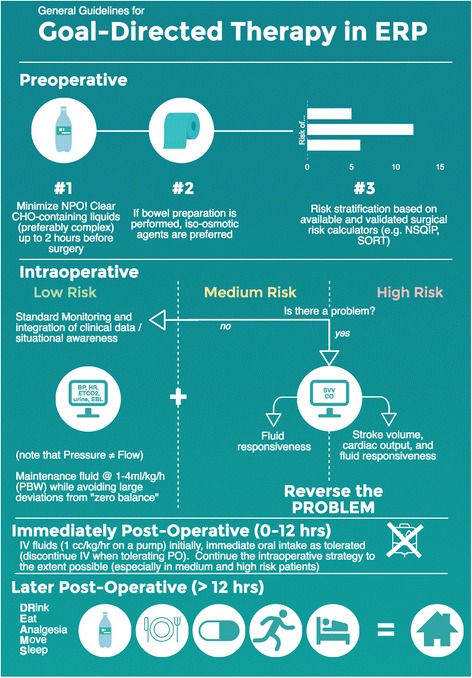
Suggested, risk-based algorithm for implementation of perioperative goal-directed in patients undergoing colorectal surgical procedures in the context of an enhanced recovery protocol

**Fig. 3:**
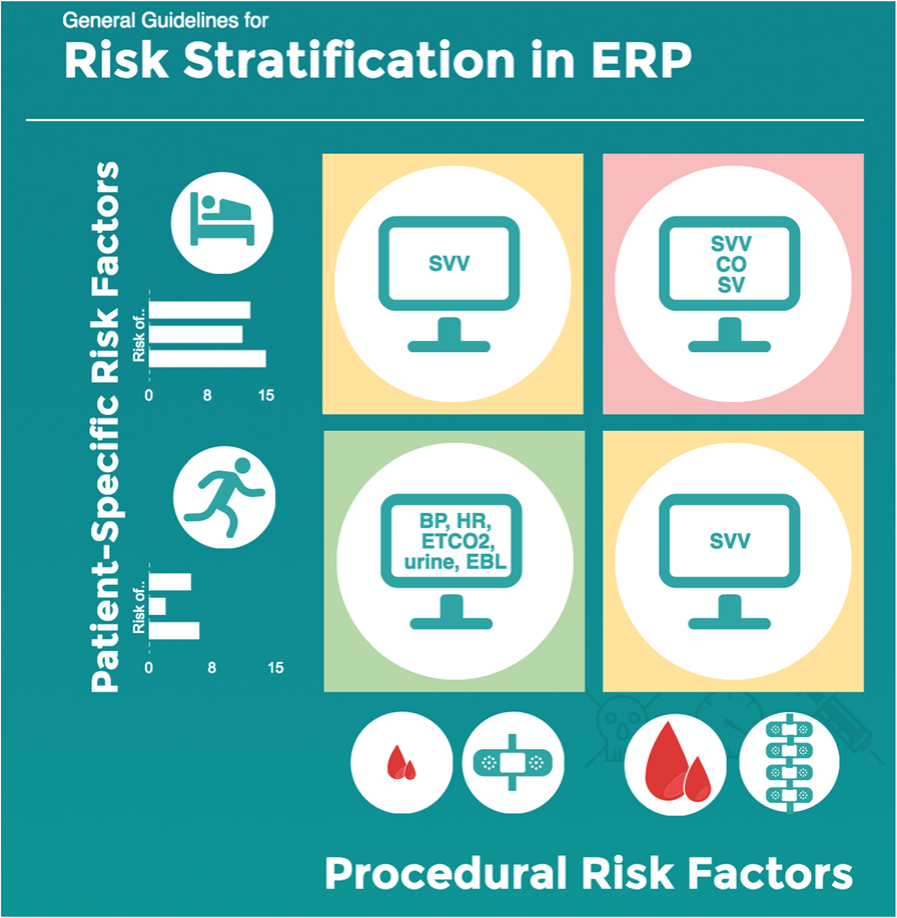
Proposed risk stratification scheme for patients undergoing colorectal surgical procedures in the context of an enhanced recovery protocol

There is little evidence that GDFT poses significant risk. The concern regarding widespread implementation is cost(s). Several investigators have examined device-guided GDFT in the modern era of ERPs. Three such groups independently tested a “zero balance” or “restrictive” strategy against conventional minimally invasive cardiac output monitoring -guided GDFT within the context of colorectal ERPs, and all found no difference in the length of stay or incident complications (335 total subjects studied) (Brandstrup et al. 2012; Srinivasa et al. 2013; Phan et al. 2014). None of these studies showed harm from GDFT. Analyzing the economic impact of esophageal Doppler-based GDFT in major abdominal surgery, but the National Institute for Health and Care Excellence (NICE) Group said that there is “cost saving per patient … when compared with the use of a central venous catheter in the perioperative period” (Excellence NIfHaC 2011). Since minimally invasive cardiac output monitoring devices are not universally used in the perioperative period, we developed a generalizable framework for perioperative fluid management incorporating such devices (Fig. 1) (Cannesson et al. 2011). Our framework does not suggest use of an algorithmic protocol (based on measures of cardiac output or surrogates) for all patients undergoing colorectal surgery; rather, we suggest that device-derived measures be placed in clinical context. For instance, fluid responsiveness rather than prompting a fluid bolus at all times may only prompt a fluid bolus when coupled with evidence of absolute hypovolemia. Abnormal physiologic values may be warning signs (not endpoints for fluid therapy), and clinical decision-making can be supplemented not replaced by the use of advanced hemodynamic monitoring devices (details are beyond the scope of this review but are discussed elsewhere) (Thiele et al. 2015b). Since intraoperative GDFT data suggests either a reduction in length of stay and complications or equipoise (albeit at higher overall cost) and also because most devices used for GDFT present minimal risk to the patient, we recommend the use of GDFT when available. We acknowledge that within ERPs for colorectal surgery, a “zero balance” approach *appears* to be an acceptable alternative; we do not recommend a simple “recipe book” fluid restriction (e.g., X ml/kg/hr) (Brandstrup et al. 2012; Srinivasa et al. 2013; Phan et al. 2014). This statement is supported by meta-analysis on GDFT within ER protocols (Rollins and Lobo 2016). The most benefit from GDFT is likely in certain subsets of patients rather than all patients undergoing colorectal surgery with ERPs. Depending on patient- and procedure-specific risks, clinicians may utilize conventional monitors or minimally invasive cardiac output monitoring devices (Fig. 2). Frameworks for three different risk categories are presented in Fig. 3.

### (v) Which fluids should be used intraoperatively?

Most intraoperative GDFT studies used colloids as the fluid of choice for volume expansion. However, colloids were also administered in the control arms of such trials thereby making it impossible to tease out the impact of colloid solutions alone (Egal et al. 2016a). It is reasonable to conclude that if colloids are going to be used, then a GDFT approach offers benefits. On the other hand, it is unclear that colloids are necessary. In a trial randomizing patients undergoing laparoscopic segmental colectomy within the context of an established ERP to three groups (with all groups receiving lactated Ringer’s at 5 mL/kg/h during the surgical procedure and anesthesia): standard fluid therapy (22 patients) versus intraoperative GDFT with lactated Ringer’s (21 patients) versus intraoperative GDFT with hetastarch (21 patients), the length of stay was longer in the GDFT groups. Furthermore, the group randomized to GDFT with lactated Ringer’s solution received the highest amount of intraoperative fluids while the group randomized to GDFT with hetastarch received the highest amount of fluids during hospitalization (Senagore et al. 2009). Yates et al. randomized 202 medium to high-risk patients undergoing colorectal surgery to receipt of a background infusion of crystalloid during the surgical procedure (1.5 mL/kg/h of Hartmann’s solution) and hemodynamic optimization (GDFT) with either Hartmann’s solution (chloride-restrictive crystalloid solution) or 6 % HES (130/0.4, Volulyte, suspended in similar solution). The results showed that there was no difference in complication rates, although the crystalloid group received more fluid than the colloid group (Yates et al. 2014). Unfortunately, there are no large, multicenter, prospective randomized controlled trials comparing crystalloid to colloid solutions intraoperatively in a GDFT protocol. Thus, when making determinations about which fluid types are most appropriate intraoperatively, clinicians are faced with either applying the results of these two small, single center trials, or extrapolating data from large crystalloid-colloid trials in unrelated populations (e.g., sepsis).

Two such trials—comparing human-derived colloids (albumin) to crystalloid in critically ill patients—found no difference in the primary outcome of 28-day mortality (Finfer et al. 2004; Caironi et al. 2014). Three trials—comparing synthetic colloids (hydroxyethyl starch solutions) to crystalloids in critically ill patients—found either an increased risk of death or an increased use of renal replacement therapy (Brunkhorst et al. 2008; Perner et al. 2012; Myburgh et al. 2012). However, synthetic colloids appeared beneficial (in terms of better survival) in an open-label trial of critically ill hypovolemic patients (Annane et al. 2013). A meta-analysis (59 randomized controlled trials with a total of 16,889 subjects) comparing crystalloids with colloids in a variety of patient populations concluded that synthetic colloids were associated with a risk of AKI and need for renal replacement therapy. Of note, subgroup analysis showed that risks were largely in patients with sepsis. General restrictions on the perioperative use of colloids are not supported by this evidence, but no sustained clinical benefits were evident with colloid use (Qureshi et al. 2016).

We therefore recommend the use of crystalloids for the treatment of hypovolemia in patients undergoing colorectal surgical procedures. We acknowledge that albumin may be safe but is more costly. We suggest that isotonic chloride-restrictive crystalloids be used based on a large body of retrospective data (Raghunathan et al. 2015; Shaw et al. 2015; Shaw et al. 2012) and some prospective trials (Shaw et al. 2015; Shaw et al. 2012). The biologic basis for such use (of chloride-restrictive buffered crystalloids over chloride-liberal solutions such as isotonic saline) is related to increased risk of hyperchloremic acidosis with the adverse pathophysiological and clinical outcomes when saline is used (Disma et al. 2014; Potura et al. 2015; Chowdhury et al. 2012; McCluskey et al. 2013; Krajewski et al. 2015; Lobo and Awad 2014). A detailed discussion of blood products is beyond the scope of this manuscript. However, we note that red blood cells have the potential for harm and blood loss should be replaced with blood products only when the risks are justified by significant anemia (Hebert et al. 1999; Hajjar et al. 2010; Carson et al. 2011).

### (vi) How do variations in surgical and anesthesia technique affect intraoperative fluid management?

The administration of fluid therapy intraoperatively depends on demonstrable “fluid responsiveness” (i.e., objective evidence that fluid therapy augments circulation). Several surgical maneuvers (e.g., Trendelenberg positioning, insufflation of the peritoneum for laparoscopy) as well as by anesthetic interventions (low tidal volume ventilation, use of positive end expiratory pressure (PEEP), utilization of thoracic epidural analgesia with local anesthetics) may impact measures of “fluid responsiveness.” Understanding the physiologic implications of these maneuvers can help clinicians contextualize changes in device-based measures and avoid the use of fluid therapy based only on the presence of “fluid responsiveness” without attendant absolute hypovolemia.

#### Trendelenberg positioning

While Trendelenberg (“head down”) positioning has been used for over a century in an effort to improve hemodynamics by augmenting venous return, its intraoperative use is primarily for better visualization of the operative site. Immediately after placing a patient in the head down position, there is a transient increase in right ventricular preload and stroke volume from increased venous blood flow (from the lower extremities and unstressed compartments). This subsequently leads to increased left ventricular output and cardiac output measurements taken 3–5 min after initiating the head down position show an increase in cardiac index (Sibbald et al. 1979). However, these changes are transient, as there is re-equilibration over time (Magder et al. 2009) and global flow returns to baseline within 10–15 min (Ostrow et al. 1994). Thus, we recommend that clinicians avoid assuming that head down positioning has a sustained benefit (long-term increase in preload) with durable improvement in intraoperative hemodynamics.

#### Laparoscopy

Peritoneal insufflation is particularly relevant in patients undergoing colorectal surgery within ERPs, since minimally invasive surgical techniques are a cornerstone of enhancing recovery. As with positioning, hemodynamic changes with the initiation of laparoscopy are transient. For instance, 5 min after initiation of a 14 mmHg pneumoperitoneum, cardiac index is significantly less than immediately prior to abdominal insufflation. However, 10 min later, there is a return to baseline (Joris et al. 1993). A recent analysis of laparoscopy to an intrabdominal pressure of 14 mmHg confirmed these findings (Alfonsi et al. 2006). It is important for clinicians to note that there is a sustained increase in mean arterial pressure with insufflation. Hence, while the shifts in blood volume (between stressed and unstressed compartments) are transient, the increased afterload (hypertension) is sustained (Joris et al. 1993; Alfonsi et al. 2006; Liu et al. 2015).

Insufflation increases the absolute value of measures such as stroke volume variation and plethysmography (Liu et al. 2015), meeting the threshold defining of “fluid responsiveness.” This does not imply that fluid therapy is needed (as discussed in question iv above) (Guinot et al. 2014). Clinicians should anticipate increases in blood pressure with minimal overall sustained changes in cardiac output (with insufflation to 14 mmHg or less). We suggest that measures of fluid responsiveness (based on cardiorespiratory interactions) are less specific after abdominal insufflation. This increase in “false positives” needs to be accounted—contextualized—as suggested in the framework we have described.

#### Low tidal volume

Lower tidal volumes confer a mortality benefit to critically ill patients with acute respiratory distress syndrome (ARDS) (Ventilation with lower tidal volumes as compared with traditional tidal volumes for acute lung injury and the acute respiratory distress syndrome. The Acute Respiratory Distress Syndrome Network. N Engl J Med 2000). The concept of “lung protective ventilation” has expanded into the operating room environment and may improve outcomes in patients (Lellouche et al. 2012; Futier et al. 2013; Severgnini et al. 2013). As cardiorespiratory interactions depend on cyclic changes in intrathoracic pressure producing corresponding cyclic changes in venous return, thresholds for “fluid responsiveness” that utilize respiratory variation in pulse pressure or systolic pressure are conditional on tidal volumes of 8–12 mL/kg (predicted body weight (PBW)) (Tavernier et al. 1998; Kramer et al. 2004). Lower tidal volumes thus increase “false negatives” decreasing arterial respiratory variation based measures of “fluid responsiveness” (Lansdorp et al. 2012; De Backer et al. 2005; Suehiro & Okutani 2011; Reuter et al. 2003). Of note, clinical trials which use respiratory variation to guide fluid management utilize tidal volumes on average of 7.8 mL/kg (range 6–9.1 mL/kg) (Benes et al. 2010; Forget et al. 2010; Ramsingh et al. 2013; Goepfert et al. 2013). We recommend that clinicians utilizing arterial (or plethysmographic) respiratory variation as a guide to “fluid responsiveness” expect a reduction in sensitivity with low tidal volume ventilation. Approaches that do not rely on such respiratory variation, such as measured response to a fluid bolus (e.g., mini-fluid challenge (Wu et al. 2014; Muller et al. 2011)), are not affected.

#### High positive end expiratory pressure

Intraoperative mechanical ventilation may now incorporate PEEP (e.g., 6–8 cm H_2_O utilized by Futier et al.) (Futier et al. 2013). Such PEEP has a hemodynamic impact as a result of increase in intrathoracic pressure (with impedance of venous return) and a decrease in left ventricular afterload (increasing the cardiac index). The overall effect is a balance between these. In general, it appears that PEEP above 5–10 cm H_2_O, leads to a decrease in cardiac index (Van Trigt et al. 1982; Terai et al. 1985; Huemer et al. 1994). Both animal and human data suggest that such decreases in cardiac index accompanying higher levels of PEEP can be reversed with fluid administration (Canfran et al. 2013; van den Berg et al. 2002; Renner et al. 2008). Furthermore, it appears that higher levels of PEEP increase dynamic indicators of fluid responsiveness (such as stroke volume variation) reducing the utility of these metrics as predictors of fluid responsiveness. We suggest that clinicians anticipate that PEEP greater than 5 cm H_2_O may decrease cardiac index, lower systemic blood pressure, and reduce the specificity of arterial (or plethysmographic) respiratory variation as guides to “fluid responsiveness.”

#### Thoracic epidural placement

The use of regional or neuraxial anesthesia is a major component of many ERPs, with thoracic epidural analgesia (TEA) most commonly used for open abdominal surgical procedures. TEA with local anesthetics can leads to hypotension from reduction in venous return (sympathectomy with venodilation) and decreases in cardiac index (Gelman et al. 1980). Arterial vasodilation also occurs (which lower systemic vascular resistance) (Goertz et al. 1992; Baron et al. 1986). Lower doses of local anesthesia have been shown to preserve cardiac index in patients receiving a TEA (Tanaka et al. 1991; Hasenbos et al. 1988). These changes induced by TEA are best thought of as shifts in internal blood volume—relative hypovolemia. Thus, low dose infusions of catecholamines will counteract these effects preserving cardiac index, and fluid therapy is not necessary (Gelman et al. 1980). We suggest that clinicians electing to use TEA recognize that hypotension may be a result of relative hypovolemia and therapy, rather than fluid administration, could be low-dose catecholamine infusions or lower rates of local anesthesia infusion (to reverse or avoid sympathectomy, respectively). It is important to point out that this recommendation is based on physiological data, not on clinical outcomes data.

### (vii) How should fluids be managed postoperatively?

Traditionally patients undergoing abdominal surgery were not allowed oral intake postoperatively, waiting for the gastrointestinal tract to “recover” before nutrition could be initiated safely (including both volume (fluids) and calories (through fluids or solid food)). In such fasting patients, replacement fluids must be provided intravenously. With ERPs a change in philosophy has occurred with surgeons often allowing oral intake immediately after surgery—provided there is no active nausea/vomiting. Given unrestricted access to oral fluids, patients can regulate their intake to preserve intravascular volume (as long as their thirst mechanisms are intact). Isolating the impact of this paradigm shift toward early postoperative oral intake is a challenge primarily because of the heterogeneity among various published colorectal ERPs. For instance, in a recent meta-analysis examining the characteristics of 13 colorectal ERPs, 9 of 13 centers allowed MBP, and only 8 of 13 centers protocolized perioperative fluid administration (Zhuang et al. 2013).

Since total fluid balance (or weight gain) after abdominal surgery is directly related to both length of stay and the incidence of complications (Brandstrup et al. 2003), it is important to avoid both postoperative hypovolemia and hypoperfusion (Bellamy 2006), as well as arbitrary infusion of intravenous fluids. The use of arterial (or plethysmographic) respiratory variation as guides to “fluid responsiveness” is challenging as patients are not mechanically ventilated after surgery and do not typically have invasive blood pressure monitoring in place (making maneuvers such as passive leg raising inapplicable). Several institutions, including Mayo Clinic, Duke University, and the University of Virginia have eliminated use of postoperative “maintenance” fluid therapy (continuous intravenous fluid administration) in colorectal patients who are able to tolerate the oral intake of clear liquids. All these sites have reported significant reductions in length of stay (Thiele et al. 2015a; Lovely et al. 2012; Miller et al. 2014). It hence seems reasonable to avoid intravenous fluid therapy when patients are tolerating clear oral liquids (often immediately after surgery).

What should be done when oral fluids are not being freely taken? In 2003, Brandstrup et al. (2003) showed that fluid balance and daily body weight are closely correlated for the first four postoperative days (Tolstrup & Brandstrup 2015) and that in patients undergoing major abdominal surgery, there is a clear relationship between total fluid balance and daily body weight gain and incident complications.

A meta-analysis has shown that maintaining patients near zero-fluid balance in the perioperative period leads to a decrease in postoperative complications with a reduction in length of hospital stay (Varadhan and Lobo 2010). We suggest that the fluid management framework utilized intraoperatively should be extended into the postoperative period, to the extent possible. In some instances, this may not be possible due to specific types of devices used during surgery. We suggest that patients tolerating clear liquids orally after surgery be given unrestricted access to such fluids and that intravenous fluid administration be avoided in this setting.

## Future research questions

1. What are the hemodynamic effects of preoperative isotonic bowel preparation (in the setting of an ERP)?
2. Is there a clinical outcome difference between simple versus complex carbohydrate loading?
3. Is a protocolized “restrictive” or “zero balance” technique equivalent to GDFT? This may be answered to some extent by the ongoing RELIEF trial (https://clinicaltrials.gov/ct2/show/NCT01424150), the focus of which is liberal versus restrictive fluid administration but which plans to examine the effect of GDFT using a statistical test of interaction. However, this study does not specifically focus on GDFT in the context of ERPs.
4. What risk stratification tool(s) best predict outcomes in patients undergoing colorectal surgery and what are the clinical and financial implications of using risk stratification to influence monitoring decisions and hemodynamic management in patients undergoing colorectal surgery?
5. Do colloids offer any benefits over crystalloid for intraoperative GDFT in non-septic patients undergoing colorectal surgery?
6. Are potential benefits of chloride-restrictive electrolyte solutions (demonstrated in retrospective analyses) demonstrable in prospective studies and are there differences within available choices of such solutions (e.g., Ringer’s lactate or Hartmann’s solution versus PlasmaLyte)?

## Summary

Iso-osmolar bowel preparation is unlikely to lead to preoperative hypovolemia requiring intravenous fluid therapy provided patients are given unrestricted access to clear fluids orally. In patients that present to the operating room in a hypovolemic state, rapid detection is feasible by dynamic indicators of fluid responsiveness such as arterial (or plethysmographic) respiratory variation. Inclusion of carbohydrates in preoperative oral fluids is likely to improve insulin sensitivity (particular when complex carbohydrates are used) and may reduce protein catabolism. Anuria is abnormal and requires immediate attention. In general, oliguria is common during and after anesthesia and surgery and should trigger diagnostic efforts but not fluid therapy until hypovolemia is established as the cause. Intraoperative fluid therapy should be based on a framework where all available information is integrated to determine if there is a physiologic problem requiring reversal. Low tidal volumes and PEEP alter the sensitivity and specificity of dynamic indicators of fluid responsiveness but have a minimal impact on cardiac index at levels commonly utilized in the operating room. To the extent possible, the approach to intraoperative fluid management should continue postoperatively.

## Abbreviations

ERP: Enhanced recovery pathway
ASER: American Society for Enhanced Recovery
POQI: Perioperative Quality Initiative
GDFT: Goal-directed fluid therapy
KDIGO: Kidney Disease: Improving Global Outcomes
MBP: Mechanical bowel preparation
CFM: Conventional fluid management
AKI: Acute kidney injury
NICE: National Institute for Health and Care Excellence
PEEP: Positive end expiratory pressure
ARDS: Acute respiratory distress syndrome
PBW: Predicted body weight
TEA: Thoracic epidural analgesia
