# Correction to: American Society for Enhanced Recovery (ASER) and Perioperative Quality Initiative (POQI) joint consensus statement on perioperative fluid management within an enhanced recovery pathway for colorectal surgery

**DOI:** 10.1186/s13741-018-0085-8

**Published:** 2018-04-10

**Authors:** Robert H. Thiele, Karthik Raghunathan, C. S. Brudney, Dileep N. Lobo, Daniel Martin, Anthony Senagore, Maxime Cannesson, Tong Joo Gan, Michael Monty G. Mythen, Andrew D. Shaw, Timothy E. Miller, For the Perioperative Quality Initiative (POQI) I Workgroup, Timothy E. Miller, Andrew D. Shaw, Michael G. Mythen, Tong J. Gan, Matthew D. McEvoy, Michael J. Scott, Deborah Gordon, Stuart Grant, Julie K. M. Thacker, Christopher L. Wu, Robert H. Thiele, Karthik Raghunathan, C. S. Brudney, Dileep N. Lobo, Daniel Martin, Anthony Senagore, Stefan D. Holubar, Traci Hedrick, John Kellum, Ruchir Gupta, Mark Hamilton, S. Ramani Moonesinghe, Mike P. W. Grocott, Elliott Bennett-Guerrero, Thomas J. Hopkins, Roberto Bergamaschi, Stuart McCluskey

**Affiliations:** 1 Departments of Anesthesiology and Biomedical Engineering, Divisions of Cardiac, Thoracic, and Critical Care Anesthesiology, UVA Enhanced Recovery after Surgery (ERAS) Program, University of Virginia School of Medicine, Charlottesville, VA USA; 2 Department of Anesthesiology, Duke University Medical Center, Durham, NC 27710 USA; 3 Duke University and Durham VA Medical Center, Durham, NC USA; 4 Gastrointestinal Surgery, National Institute for Health Research Nottingham Digestive Diseases Biomedical Research Unit, Nottingham University Hospitals and University of Nottingham, Queen’s Medical Centre, Nottingham, NG7 2UH UK; 5 Division of Surgery and Interventional Science, University College London, Royal Free Hospital, London, NW3 2QG UK; 6 Anaesthetic Department, Royal Free Perioperative Research Group, Royal Free Hospital, London, NW3 2QG UK; 7 Department of Surgery, University of Texas-Medical Branch at Galveston, Galveston, TX 77555 USA; 8 Department of Anesthesiology and Perioperative Medicine, University of California Los Angeles, Los Angeles, CA USA; 9 Department of Anesthesiology, Stony Brook University School of Medicine, Stony Brook, NY USA; 10 University College London Hospitals, National Institute of Health Research Biomedical Research Centre, London, UK; 11 Department of Anesthesiology, Vanderbilt University School of Medicine, Nashville, TN USA; 12 Division of General, Vascular and Transplant Anesthesia, American Society for Enhanced Recovery, Duke University Medical Center, Durham, NC 27710 USA

## Correction

After publication of this article (Thiele et al., 2016), it was noticed that the HTML version contained errors with the collaborator group, the Perioperative Quality Initiative (POQI) I Workgroup, preventing the group members from being searchable within indexing repositories. The corrected declarations section can be found here.
